# RETRACTION: Quercetin Preserves *β*-Cell Mass and Function in Fructose-Induced Hyperinsulinemia through Modulating Pancreatic Akt/FoxO1 Activation

**DOI:** 10.1155/ecam/9836162

**Published:** 2025-08-27

**Authors:** Evidence-Based Complementary and Alternative Medicine

RETRACTION: J.-M. Li, W. Wang, C.-Y. Fan, et al., “Quercetin Preserves *β*-Cell Mass and Function in Fructose-Induced Hyperinsulinemia through Modulating Pancreatic Akt/FoxO1 Activation,” *Evidence-Based Complementary and Alternative Medicine* 2013 (2013): 303902, https://doi.org/10.1155/2013/303902.

The above article, published online on 27 February 2013 in Wiley Online Library (https://wileyonlinelibrary.com), has been retracted by John Wiley & Sons Ltd.

The retraction has been agreed following an investigation of the concerns raised by *Lecithocera eumenopis* on PubPeer [1], which identified several instances of similarity between the western blot bands in the following figures:

- Figure 2a: The Pdx1 bands are similar to the Nuclear Pdx1 bands when horizontally flipped in Figure 4b.

- Figures 5a-b: The western blots for Akt appear overlapping despite different experimental conditions.

Inconsistencies have also been identified regarding the description of the number of animals reported in each group throughout the experiment.

As a result of the investigation, the data and conclusions of this article are considered unreliable.

The authors disagree with this retraction.
